# Evaluating the efficacy and acceptability of vagus nerve stimulation for fibromyalgia: a PRISMA-compliant protocol for a systematic review and meta-analysis

**DOI:** 10.3389/fneur.2024.1367295

**Published:** 2024-02-21

**Authors:** Yunhuo Cai, Yajun Zhang, Yiyan Fang, Hantong Hu, Xingling Li, Lianqiang Fang

**Affiliations:** ^1^Department of Rehabilitation, The Third Affiliated Hospital of Zhejiang Chinese Medical University, Hangzhou, China; ^2^The Third Clinical Medical College, Zhejiang Chinese Medical University, Hangzhou, China; ^3^Department of Acupuncture and Moxibustion, The Third Affiliated Hospital of Zhejiang Chinese Medical University, Hangzhou, China

**Keywords:** fibromyalgia, vagus nerve stimulation, protocol, efficacy, acceptability

## Abstract

**Background:**

Fibromyalgia has imposed substantial burdens on patients’ health and well-being, yet effective therapeutic options for this condition remain limited. Recently, vagus nerve stimulation (VNS) has emerged as a promising therapy for fibromyalgia. Nonetheless, despite the increasing number of randomized clinical trials (RCTs), current evidence remains inconclusive. Therefore, this protocol of a systematic review and meta-analysis aims to synthesize the existing evidence to clarify the efficacy and acceptability of VNS for treating fibromyalgia.

**Methods:**

A comprehensive search for eligible RCTs will be conducted across nine bibliographic databases, namely PubMed, Cochrane Library, Embase, AMED, PsycINFO, PEDro, Chinese BioMedical Literature Database, Chinese National Knowledge Infrastructure, and Wangfang database. Data obtained from the included studies will be synthesized quantitively using RevMan 5.4.1 for meta-analyses. The methodological soundness of included RCTs will be assessed via the Cochrane’s updated risk of bias tool (version 2.0). Additionally, sensitivity analyses, publication bias assessment, and subgroup analyses will be conducted as appropriate. Finally, we will utilize the Grading of Recommendations, Assessment, Development, and Evaluation (GRADE) system to evaluate the certainty for the body of evidence.

**Conclusion:**

The findings of our study are anticipated to ascertain the efficacy and acceptability of VNS as a promising treatment option for fibromyalgia. This will not only fill current research gap but also identify potential areas for future research. The findings will provide essential guidance for evidence-based treatment decisions for fibromyalgia, benefiting both patients and clinicians.

## 1 Introduction

Fibromyalgia stands as a predominant chronic pain condition, characterized by widespread pain, fatigue, sleep disturbances, cognitive dysfunction, and depression (1). Its pathogenesis is linked to central sensitization, in addition to factors such as inflammation, genetics, and psychosocial influences (2). The prevalence of fibromyalgia exhibits significant geographical disparities, with rates ranging from under 1% in Denmark to 2.4% in Spain, and from 2.0 to 3.3% in North America (3). Furthermore, the condition shows a distinct gender difference, being more prevalent among women than men, and it also demonstrates an increasing trend with advancing age.

Pharmacological interventions, such as duloxetine and pregabalin, are frequently utilized in the management of fibromyalgia, providing pain relief and enhancing overall patient functioning (4). Nonetheless, pharmacotherapy for fibromyalgia can have certain side effects associated with medication. Additionally, given that fibromyalgia is a complex condition with both physical and psychological components, patients can be benefit from non-pharmacological approaches such as mindfulness meditation, body awareness therapies, and exercise therapy (5), which address various facets of the disorder and improve overall well-being. These non-pharmacological therapies can potentially reduce the polypharmacy burden, provide symptom relief with fewer side effects compared to medication, and improve overall well-being. Furthermore, non-pharmacological treatments allow patients to actively manage their condition, influencing lifestyle factors like stress levels, and when combined with pharmacological interventions, may provide additional benefits for patients. However, despite the numerous available therapies, a noteworthy proportion of patients exhibit a lack of active response to them. To date, there has been a lack of therapies with widely recognized efficacy for fibromyalgia. As a result, there is an urgent need for alternative therapies that offer favorable efficacy, safety, and good tolerance (6).Vagus nerve stimulation (VNS) has emerged as a promising technique in the field of neuromodulation. It is indicated for the treatment of various diseases across multiple systems, encompassing neurological conditions (e.g., headache, migraine, tinnitus), psychological disorders (e.g., depression, anxiety, insomnia), cardiovascular diseases (e.g., hypertension, heart failure), and gastrointestinal disorders (e.g., inflammatory bowel disease, functional dyspepsia) (7). Notably, the Food and Drug Administration (FDA) granted approval for VNS in the treatment of refractory epilepsy in 1997 and resistant depression in 2005. The application of VNS can be broadly categorized into two forms: invasive, which involves the implantation of stimulating electrodes at the cervical branch of the vagus nerve, and noninvasive, which employs transcutaneous modalities. When electrical stimulation is specifically applied to the auricular branch of the vagus nerve that is distributed in the concha or the lower half of the back ear, it is referred to as transcutaneous auricular vagus nerve stimulation (ta-VNS) (8, 9). ta-VNS has demonstrated a comparable modulatory effect to the invasive vagus nerve stimulation (i-VNS), while exhibiting a more favorable safety profile and ease of operation (10). In recent decades, VNS has emerged as a promising new approach for treating various kinds of chronic pain conditions such as fibromyalgia, as it has been shown to reduce pain by modulating descending serotonergic and noradrenergic neurons (11). These neurons play a pivotal role in central sensitization, a process closely correlated with the pathogenesis of fibromyalgia.

### 1.1 The feasibility and significance of conducting this study

To note, the feasibility and significance of conducting this systematic review (SR) and meta-analysis can be highlighted as follows. In recent years, there has been a notable rise in clinical trials, including randomized controlled trials (RCTs), aimed at investigating the therapeutic effect of VNS on fibromyalgia. For example, Lange et al. (12) conducted a trial to explore the efficacy and tolerance of i-VNS in patients with fibromyalgia. The results demonstrated a favorable efficacy and tolerance to i-VNS treatment. Another RCT (13) conducted in 2020 investigated the efficacy of ta-VNS in combination with exercise on 60 patients with fibromyalgia, ultimately leading to significantly reduced pain intensity. However, the comparison between the treatment group, which received ta-VNS combined with exercise, and the control group, which solely underwent exercise, revealed no statistically significant between-group differences. In addition, a more recent RCT (14) published in 2022 sought to compare the therapeutic effects of ta-VNS, sham ta-VNS, and meditation-based diaphragmatic breathing for treating fibromyalgia. The results of this RCT revealed significant inter-group differences in overall fibromyalgia severity, while it did not manifest noteworthy differences in average pain intensity across different groups. Furthermore, there are several ongoing RCTs or trials (15–17) with upcoming results to be published. Despite the increasing number of trials in this field, however, the current body of literature exhibits a dearth of accessible SRs and meta-analyses concerning the efficacy and acceptability of VNS for fibromyalgia treatment, thus hindering the establishment of definitive conclusions. Consequently, our study aims to address this research gap by contributing valuable insights in this area. Moreover, the publication of the aforementioned trials makes it feasible to perform our study because it is expected to incorporate a sufficient number of eligible trials into the SR and meta-analysis. As discussed above, the significance and feasibility of this SR and meta-analysis are well-founded.

### 1.2 Research objective

Since that the review question for our SR and meta-analysis is whether VNS is effective for fibromyalgia with favorable acceptability, the objective of our study is to systematically synthesize the evidence concerning the efficacy and acceptability of VNS in fibromyalgia.

## 2 Methods

Our present study protocol is carefully designed and reported based on the guideline of the Preferred Reporting Items for Systematic Reviews and Meta-analysis Protocols (PRISMA-P) (18) (as shown in Supplementary material).

### 2.1 Prospective registration

This reported protocol is specified in advance and registered in the International Prospective Register of Systematic Reviews (PROSPERO) platform in advance with the identification number CRD42023449232.

### 2.2 Eligibility criteria for included studies

The criteria for the inclusion of eligible studies are meticulously designed according to the “*PICOS*” (i.e.; *P* for participants, *I* for interventions, *C* for controls, *O* for outcome measures, and *S* for study designs) framework.

#### 2.2.1 Participants

The eligibility criteria for participants are adults (aged >18 years) who have a definite diagnosis of fibromyalgia using one of the widely accepted criteria built by the American College of Rheumatology [versions published in 1990 (19), 2010 (20) or 2016 (21)].

#### 2.2.2 Interventions

Eligible interventions will be restricted to VNS, which is performed via placement of the vagus nerve stimulator. As the efficacy of VNS for fibromyalgia is undetermined, the intervention group’s eligibility criteria will be limited to the use of VNS alone. VNS combined with other active interventions will not be considered in this context. Alone No limitations will be imposed on the types (e.g., invasive, or noninvasive VNS), stimulator models, and stimulus parameters of VNS.

#### 2.2.3 Comparisons

1. No intervention or waiting list control;
2. Placebo/sham controls (e.g., sham VNS, placebo medication).

#### 2.2.4 Outcome measures

Drawing from references to similar SRs and meta-analysis studies in this field (22–24), our study focuses on several key outcome measures. These measures include pain intensity, fatigue, sleep quality, psychological disorders, quality of life, and patient acceptability. To be considered for inclusion in our study, a study must report at least one of the above-mentioned outcome measures.

##### 2.2.4.1 Primary outcome

The primary outcome is pain intensity measured by the Visual Analog Scale (VAS) and/or other validated instruments such as Numeric Rating Scale (NRS), Verbal Rating Scale (VRS), Brief Pain Inventory (BPI), and short-form McGill Pain Questionnaire (SF/MPQ).

##### 2.2.4.2 Secondary outcomes

###### 2.2.4.2.1 Fatigue

Fatigue associated with fibromyalgia can be measured by standardized scales, such as Multidimensional Fatigue Inventory (MFI), Fatigue Severity Scale, and Multidimensional Assessment of Fatigue Global Index.

###### 2.2.4.2.2 Sleep quality

The assessment of sleep quality can be effectively accomplished using self-reported scales that have demonstrated both reliability and validity (e.g., the Pittsburgh Sleep Quality Index (PSQI), the Insomnia Severity Index (ISI)).

###### 2.2.4.2.3 Psychological disorders

Psychological disorders (e.g., depression, anxiety) associated with fibromyalgia can be measured by standardized questionnaires, for examples, Beck Depression Inventory (BDI) and Hamilton Depression Scale (HAMD) for evaluating depression, and Self-Assessment Scale for Anxiety (SAS) for evaluating anxiety.

###### 2.2.4.2.4 Quality of Life (QoL)

QoL can be evaluated by multiple standardized scales, such as the Fibromyalgia Impact Questionnaire (FIQ), the General Health Questionnaire (GHQ), and the Short Form 36 Health Survey (SF-36).

###### 2.2.4.2.5 Patient acceptability

Patient acceptability is characterized by the number of discontinuations attributed to adverse reactions associated with VNS or the control treatment. This outcome will be quantified by the proportion of participants who withdraw from the trial for any reason, relative to the total number of patients initially randomly assigned to each group.

#### 2.2.5 Study designs

The eligible type of studies will be restricted to RCTs. Additionally, only studies published in English and Chinese language will be included due to the research team’s inability to finance translation services for other foreign languages. Studies will be excluded if they are non-RCTs, trials without a control group, utilize comparators that not included in the above predetermine list, or contain fewer than five participants in any treatment arm.

### 2.3 Identification of eligible studies and search strategy

#### 2.3.1 Identification of studies via databases and registry platforms

A comprehensive search will be performed across the subsequent bibliographic databases: PubMed, Cochrane Library, Embase, AMED, PsycINFO, PEDro, Chinese BioMedical Literature Database (CBM), Chinese National Knowledge Infrastructure (CNKI), and Wangfang database. Potentially eligible publications in these databases will be searched from inception to 31 December, 2023. Additionally, an add-on search will be conducted by thoroughly searching five prominent clinical trial registry platforms: the ClinicalTrials.gov registry, the International Clinical Trial Registration Platform (ICTRP), the Chinese Clinical Trial Registry, the Australian New Zealand Clinical Trials Registry (ANZCTR), and the ISRCTN registry. Our aim is to identify ongoing trials containing unpublished data relevant to this research topic. We will then contact the principal investigators of these ongoing trials via email to request the most recent data.

To ensure a comprehensive search for eligible studies, we will employ a well-established and rigorous search strategy. This strategy will include a combination of subject headings, such as medical topic heading words (MeSH) for PubMed, and relevant free text terms related to fibromyalgia, vagus nerve stimulation, and randomized controlled trials. Our planned search will not be limited by terms relating to the types of control interventions used, thereby ensuring the inclusion of all relevant studies comparing the effectiveness of VNS therapy against any standard treatment or management option for fibromyalgia.

For the search strategy in PubMed, a detailed illustration is provided in Table 1. Adapting this search strategy to the remaining bibliographic databases will entail substituting MeSH terms with relevant subject headings (when available), while maintaining consistency with the use of relevant free text terms. The detailed search strategies for the other databases were provided in the Supplementary material.

**Table 1:** Search strategy in PubMed.

No.	Search items
#1	Randomized controlled trial [Publication type]
#2	Controlled clinical trial [Publication type]
#3	Random* [Title/Abstract]
#4	Clinical trials [MeSH]
#5	Randomly [Title/Abstract]
#6	RCT [Title/Abstract]
#7	#1 OR #2 OR #3 OR #4 OR #5 OR #6
#8	Humans [MeSH]
#9	#7 AND #8
#10	Fibromyalgia [MeSH] OR Fatigue Syndrome, Chronic [MeSH]
#11	Fibromyalgia*[Title/Abstract] OR Muscular Rheumatism [Title/Abstract] OR Fibrositis [Title/Abstract] OR Diffuse Myofascial Pain Syndrome [Title/Abstract]
#12	#10 OR #11
#13	Vagus Nerve Stimulation [MeSH] OR Vagus Nerve [MeSH]
#14	VNS [Title/Abstract] OR taVNS [Title/Abstract] OR ta-VNS [Title/Abstract] OR iVNS [Title/Abstract] OR vagus nerve stimulation [Title/Abstract]
#15	#13 OR #14
#16	#9 AND #12 AND #15

#### 2.3.2 Identification of studies through supplementary approaches

To expand the potential pool of studies, we will conduct thorough examinations of the reference lists of all identified publications to supplement any additional eligible studies. Furthermore, we will perform comprehensive searches and browse through relevant associations, institutions, and preprint servers to uncover any supplementary trials. Specifically, we will include preprint studies from reputable platforms such as Research Square,^1^ medRxiv,^2^ and Arxiv.^3^ Furthermore, we will explore grey literature, which includes conference proceedings, academic dissertations, and government reports, as potential sources of valuable information. The incorporation of these supplementary approaches is intended to enhance the comprehensiveness and robustness of our study selection process.

### 2.4 Data acquisition and analysis

#### 2.4.1 Study selection process

**Figure 1:**
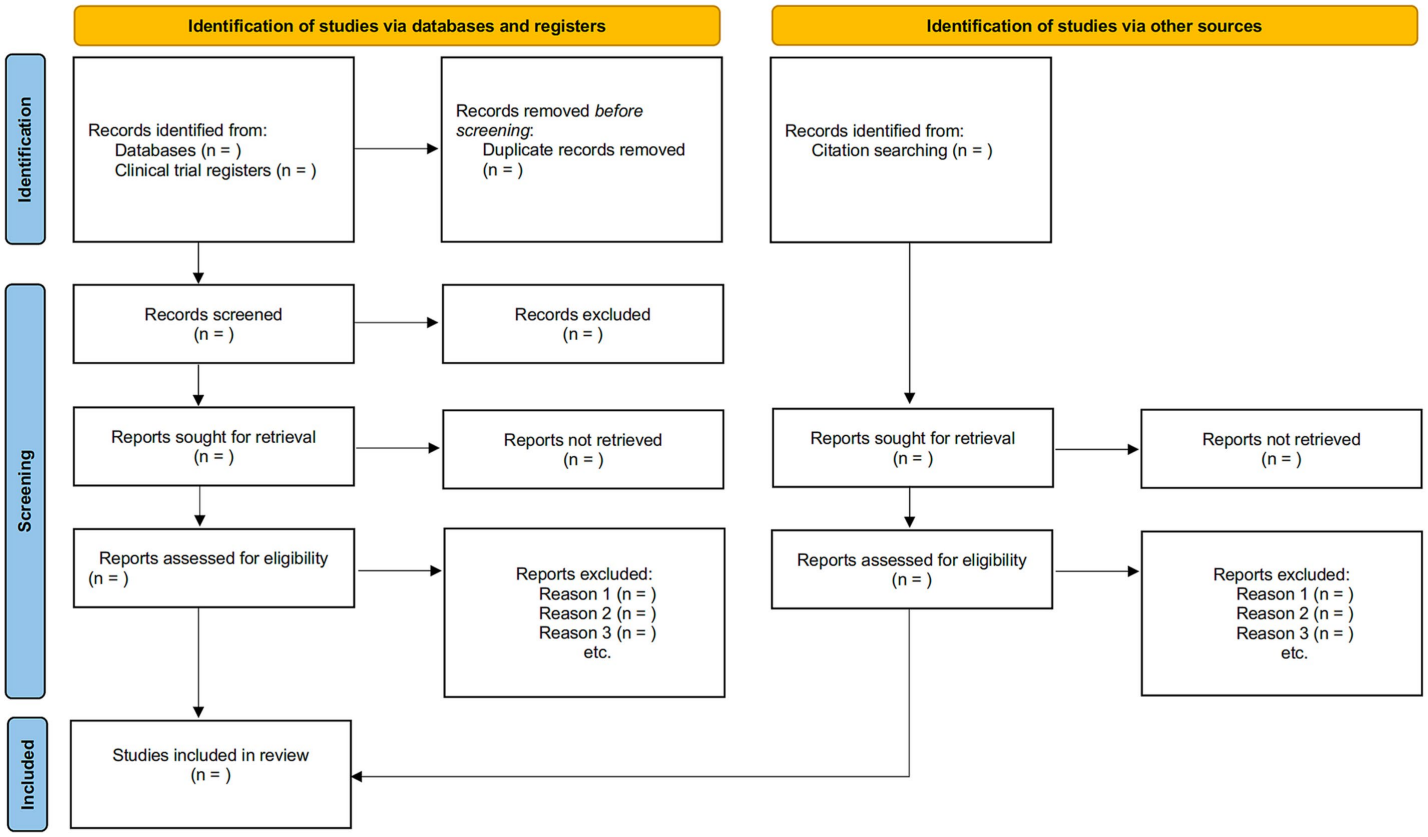
Flow diagram of study selection.

The inclusion and exclusion criteria will be applied to determine potential eligible studies by screening the titles and abstracts of corresponding papers initially, followed by a full-text screening by two independent reviewers. Should discrepancies arise, a third senior reviewer will arbitrate such conflicts. The illustration outlining the procedure for study selection is presented in the PRISMA flowchart (Figure 1).

#### 2.4.2 Data collection and management

Upon inclusion of all eligible studies, two independent extractors will use a standardized extraction form to collect relevant data and information. The extracted data will be carefully cross-checked for accuracy. The standardized extraction form will mainly encompass demographic and clinical characteristics, including study design, details of the study population, types of VNS (e.g., invasive, noninvasive), stimulation parameters of VNS (e.g., stimulation frequency, pulses, intensity), types of comparator interventions, treatment frequency and durations, outcome measurements, and other pertinent details. For continuous data in each group of the original trial, the mean value and standard deviation (SD) will be extracted along with the total number of participants. When continuous data are reported as median (range) and/or median (interquartile range (IQR)), we first contact the study authors to request the mean (SD) data. If no reply is obtained from the authors, we will adopt the preferred method described by Wan et al. (25) to convert median, IQR, and range values into mean (SD), which is based on a sample size-dependent extension of the formula for approximating the SD using IQR or range values. For dichotomous data, the number of respondents and the total number of participants in each category will be recorded.

In addition, assuming that crossover RCTs are included in the review, we will only extract and analyze the preliminary results from the two groups prior to the crossover by referencing to related studies. In addition, data obtained from three-arm RCTs included in the meta-analysis will undergo processing. This will involve the division of the common group found within multi-arm trials, allowing for a pairwise meta-analysis approach, which is a widely recognized method (26). Any discrepancies that may arise during the selection of RCTs and data extraction will be resolved through discussion or arbitration, facilitated by a senior researcher.

#### 2.4.3 Handling of missing data

In cases where essential data is not accessible within the original publication of the included trial, we will contact the corresponding author of the original study via email and make reasonable requests for the relevant data.

#### 2.4.4 Assessment of the methodological quality of included studies

To evaluate the methodological quality of each RCT, two independent raters will use the Cochrane’s updated risk of bias (ROB) tool (version 2.0) (27). The ROB assessment will focus on five critical aspects: (1) randomization process, (2) handling of missing data for outcomes, (3) deviations from intended interventions, (4) outcome assessment, and (5) selection of study results. Each ROB item will be categorized as “low,” “high” or “some concerns” (27). Additionally, an overall ROB rating for each study will be assigned as “low” (indicating low ROB for all items), “some concerns” (indicating some concerns in at least one item), or “high” (indicating high ROB for at least one item or some concerns for various items). In case of any discrepancies between the two raters, they will be resolved through negotiations with a senior reviewer.

#### 2.4.5 Treatment effect measures

To combine and analyze treatment effect data, we will utilize the meta-analysis statistical program RevMan Version 5.4.1. For continuous data, we will compute the weighted mean difference or standardized mean difference, accompanied by their corresponding 95% confidence intervals (CI). Dichotomous data will be analyzed using the risk ratio with the corresponding 95% CI.

#### 2.4.6 Evaluation of heterogeneity across studies

When evaluate the heterogeneity across the included studies, we will employ the Cochran Q-test and I2 statistic, which expresses the percentage of total variability attributed to between-study heterogeneity. If the Q-test yields a *p*-value greater than 0.10 and the I2-value is below 50%, indicating an acceptable level of heterogeneity, a fixed-effect model will be adopted for the quantitative analysis of pooled data (28). However, if the Q-test’s p-value is equal to or less than 0.10 and the I2-value is 50% or higher, indicating significant heterogeneity, the random-effect model will be employed (28). Furthermore, when applicable, subgroup analyses will be conducted to investigate potential sources of significant heterogeneity.

#### 2.4.7 Data synthesis

Data synthesis using meta-analysis (i.e., quantitative analysis) will be undertaken in the RevMan software (Version 5.4.1). Notably, a descriptive qualitative description (i.e., qualitative analysis) of the findings will be provided if the clinical and methodological heterogeneity makes it impractical to conduct the meta-analysis.

#### 2.4.8 Subgroup analysis

When applicable, we will conduct subgroup analyses to investigate possible sources of significant heterogeneity based on the following characteristics of the original studies.

1. Variations in stimuli parameters (e.g., stimulation frequency, pulses, duration) of ta-VNS.
2. Different measurement timepoints for primary outcomes (e.g., short-term effect vs. long-term effect).

#### 2.4.9 Sensitivity analysis

To ensure the rigor of the meta-analysis results and assess the impact of individual studies on the overall effect size, a sensitivity analysis will be performed using the established leave-one-out approach. If inconsistent results are identified during sensitivity analysis, the results of the corresponding meta-analyses will be interpreted with great caution.

#### 2.4.10 Assessment of certainty of evidence

The certainty of evidence produced by this SR and meta-analysis will be independently evaluated by two raters using the Grading of Recommendation, Assessment, Development, and Evaluation (GRADE) framework (29). Various factors, such as research constraints, inconsistent results, indirectness of evidence, reporting bias, and imprecision, will be taken into consideration when determining the certainty of evidence. To ensure accuracy, the outcomes of the GRADE evaluation are subject to verification, with the resolution of any discrepancies being carried out by a senior researcher.

#### 2.4.11 Evaluation of publication Bias

To investigate potential publication bias and the influence of studies with small sample sizes, we will employ a funnel plot. For meta-analysis results with more than 10 trials included, we will assess the asymmetry of the funnel plot using Begg’s and Egger’s tests (28). A *p*-value less than 0.05 in these tests will indicate a significant level of publication bias.

### 2.5 Patient and public involvement

Patients or the public will not be involved in the development of the research topic, methodology design, data collection, outcome measurements, or data analysis of this study. Consequently, the study participants will not receive the dissemination of the study’s results.

## 3 Discussions

Drawing from previous literature search, our study reports the first SR and meta-analysis protocol regarding efficacy and safety of VNS on fibromyalgia, which is an important topic for clinical practice. As a neuromodulation technique with significant application value, VNS is frequently used for a variety of diseases, mainly including neurological disorders, pain conditions, and gastrointestinal disorders (30). Regarding the relevant mechanisms of action, VNS has the capacity to modulate vagal activity and neuro-immune communication, thereby achieving analgesic effects in addition to ameliorating neurological disorders, as indicated by findings from both human and animal studies (11). In addition, VNS exerts its influence on numerous brain regions involved in pain processing, thus presenting a promising avenue for pain modulation. Furthermore, the anti-inflammatory properties of VNS may also contribute significantly to its pain-inhibitory effects. Therefore, VNS has been increasing utilized to treat a wide range of diseases, such as chronic pain conditions (11, 31), encompassing fibromyalgia (14), visceral pain (32), rheumatoid arthritis (33), trigeminal allodynia (34), and migraine (35, 36). To note, clinical trials (12–14) published in recent years reveal that patients with fibromyalgia can benefit from VNS, thereby indicating that VNS may be a promising therapy for fibromyalgia.

However, despite the increasing number of published studies and ongoing clinical trials in this field, it is noteworthy that the therapeutic effect and acceptability of VNS in the treatment of fibromyalgia remain inconclusive due to the lack of published SR and meta-analysis. Therefore, there is a need for conducting a SR and meta-analysis study to comprehensively examine the evidence and determine the effectiveness and safety of VNS for fibromyalgia. Considering the research gaps, we have meticulously designed the current PRISMA-compliant protocol to provide a thorough outline of the rationale, feasibility, and methodological procedures for conducting the subsequent SR and meta-analysis on this clinically significant topic.

### 3.1 Strengths of the current study

First, our study addresses the challenge posed by the lack of well acknowledged therapies for fibromyalgia, both pharmacological and non-pharmacological. Consequently, fibromyalgia remains a difficult disease to treat. Therefore, establishing robust evidence on the efficacy and patient acceptability of VNS for fibromyalgia will offer valuable guidance to clinicians, patients, and policymakers. This evidence will help determine whether VNS, as a stand-alone treatment or an adjunct, could be a viable therapeutic approach for treating fibromyalgia, thereby enhancing current treatment strategies. Based on comprehensive literature search in advance, to the best of our understanding, our study is the first SR and meta-analysis protocol that aims to synthesize evidence regarding the efficacy and acceptability of VNS in alleviating fibromyalgia, following a comprehensive literature search.

Second, our investigation will assess the quality of evidence through the utilization of the GRADE methodology (29). The outcomes of the systematic review will captivate a wide-ranging readership, encompassing individuals diagnosed with fibromyalgia, healthcare practitioners involved in its management, and insurers/compensation boards. The results and findings derived from our study will contribute to the refinement of evidence-based management of fibromyalgia and identify essential areas warranting further investigation.

Third, this protocol has been meticulously developed in adherence to the authoritative PRISMA-P guidelines (18) and is prospectively registered in the validated PROSPERO platform. These measures ensure the overall methodological quality of the subsequent completed SR and meta-analysis, enhance research transparency, and minimize potential performance bias (37).

### 3.2 Limitations

First, the limited affordability of translation services for various languages restricts the publication language to Chinese and English. This constraint might introduce a selection bias in the inclusion of studies. Second, given that there is currently no consensus on the optimal treatment protocols for VNS, such as stimulus sites and parameters. The varied treatment approaches utilized in the included trials may increase the level of heterogeneity, potentially hindering the effectiveness of quantitative analysis. In such situations, if appropriate, an alternative approach using descriptive qualitative accounts of the findings will be considered.

## 4 Conclusion

This protocol outlines the rationale, feasibility and methodology for a SR and meta-analysis that intends to synthesize the existing evidence pertaining to the use of VNS in the treatment of fibromyalgia. The findings are expected to ascertain the efficacy and acceptability of VNS in alleviating fibromyalgia, which will not only clarify the current state of evidence but also highlight any existing gaps. Ultimately, these findings will provide valuable insights that assist both patients and clinicians in making well-informed and appropriate treatment decisions for fibromyalgia.

## Author contributions

YC: Investigation, Writing – original draft. YZ: Writing – original draft. YF: Writing – original draft. HH: Methodology, Writing – review & editing. XL: Writing – review & editing. LF: Conceptualization, Validation, Writing – review & editing.

## Footnotes

^1^ www.researchsquare.com/

^2^ www.medrxiv.org/

^3^ www.arxiv.org/

## Glossary

ACR: American College of Rheumatology
BDI: Beck Depression Inventory
BPI: Brief Pain Inventory
CI: Confidence Interval
FDA: Food and Drug Administration
FIQ: Fibromyalgia Impact Questionnaire
GHQ: General Health Questionnaire
GRADE: Grading of Recommendation, Assessment, Development, and Evaluation
HAMD: Hamilton Depression Scale
IQR: Interquartile Range
i-VNS: Invasive Vagus Nerve Stimulation
ISI: Insomnia Severity Index
MeSH: Medical Subject Headings
MFI: Multidimensional Fatigue Inventory
NRS: Numeric Rating Scale
PRISMA-P: Preferred Reporting Items for Systematic Reviews and Meta-Analysis Protocols
PSQI: Pittsburgh Sleep Quality Index
QoL: Quality of Life
RCT: Randomized Controlled Trial
ROB: Risk of Bias
SAS: Self-Assessment Scale for Anxiety
SD: Standard Deviation
SF-36: Short Form 36 Health Survey
SF/MPQ: Short-form McGill Pain Questionnaire
SR: Systematic Review
ta-VNS: Transcutaneous Auricular Vagus Nerve Stimulation
VAS: Visual Analog Scale
VNS: Vagus Nerve Stimulation
VRS: Verbal Rating Scale
