# Supplementary material for: Evaluating the efficacy and acceptability of vagus nerve stimulation for fibromyalgia: a PRISMA-compliant protocol for a systematic review and meta-analysis
Source: Front Neurol. 2024 Feb 21;15:1367295. doi: 10.3389/fneur.2024.1367295 (PMC10914987; doi:10.3389/fneur.2024.1367295)
Supplement: Supplementary file 1 [file Table_1.DOCX]

**Supplementary Materials: Search strategies in multiple databases**

1. **Cochrane Library**

#1 (Randomized controlled trial OR Controlled clinical trial) [Publication Type]

#2 "Random* [Title/Abstract/Keywords]

#3 "RCT" [Title/Abstract/Keywords]

#4 #1 OR #2 OR #3

#5 Fibromyalgia [Title/Abstract] OR Muscular Rheumatism [Title/Abstract] OR Fibrositis [Title/Abstract] OR Diffuse Myofascial Pain Syndrome [Title/Abstract/Keywords]

#6 Vagus Nerve Stimulation OR VNS OR taVNS OR iVNS [Title/Abstract/Keywords]

#7 #4 AND #5 AND #6

1. **Embase (via Ovid)**

#1 'randomized controlled trial'/exp OR 'controlled clinical trial'/exp

#2 random*:ab,ti OR RCT:ab,ti

#3 #1 OR #2

#4 'human'/exp

#5 #3 AND #4

#6 'fibromyalgia'/exp OR 'chronic fatigue syndrome'/exp

#7 'vagus nerve stimulation'/exp

#8 #6 AND #7

#9 #5 AND #8

1. **AMED**

#1 Random*.mp. OR RCT.mp.

#2 Fibromyalgia.mp. OR Chronic Fatigue Syndrome.mp.

#3 Vagus Nerve Stimulation.mp. OR “VNS”.mp. OR “taVNS”.mp. OR “iVNS”.mp.

#4 #1 AND #2 AND #3

1. **PsycINFO**

#1 Random* OR controlled [Abstract /Title/Keywords]

#2 RCT [Abstract /Title/Keywords]

#3 #1 OR #2

#4 Fibromyalgia OR Muscular Rheumatism OR Fibrositis OR Diffuse Myofascial Pain Syndrome [Abstract /Title/Keywords]

#5 vagus nerve stimulation OR VNS OR taVNS OR ta-VNS OR iVNS [Abstract/Title/Keywords]

#6 #3 AND #4 AND #5

1. **PEDro**

#1 Fibromyalgia OR Muscular Rheumatism OR Fibrositis OR Diffuse Myofascial Pain Syndrome [Abstract/Title]

#2 vagus nerve stimulation OR VNS OR taVNS OR ta-VNS OR iVNS [Abstract/Title]

#3 clinical trial [Method]

#4 #1 AND #2 AND #3

1. **Chinese National Knowledge Infrastructure (CNKI)**

#1 随机 [标题/摘要]

#2 对照 [标题/摘要]

#3 #1 OR #2

#4 纤维肌痛 OR 纤维肌痛综合征 OR纤维肌痛症 OR 弥散性肌痛 OR 弥漫性肌筋膜疼痛 OR 弥漫性肌筋膜疼痛综合症 [标题/摘要]

#5 迷走神经刺激 OR 迷走神经 [标题/摘要]

#6 VNS [标题/摘要] 或 taVNS [标题/摘要] 或 ta-VNS [标题/摘要] 或 iVNS [标题/摘要]

#7 #5 OR #6

#8 #3 AND #4 AND #7

1. **Wangfang Database**

#1 随机 [标题/摘要]

#2 对照 [标题/摘要]

#3 #1 OR #2

#4 纤维肌痛 OR 纤维肌痛综合征 OR纤维肌痛症 OR 弥散性肌痛 OR 弥漫性肌筋膜疼痛 OR 弥漫性肌筋膜疼痛综合症 [标题/摘要]

#5 迷走神经刺激 OR 迷走神经 [标题/摘要]

#6 VNS [标题/摘要] 或 taVNS [标题/摘要] 或 ta-VNS [标题/摘要] 或 iVNS [标题/摘要]

#7 #5 OR #6

#8 #3 AND #4 AND #7

1. **Chinese BioMedical Literature Database (CBM)**

#1 随机

#2 对照

#3 #1 OR #2

#4 纤维肌痛 OR 纤维肌痛综合征 OR 纤维肌痛症 OR 弥散性肌痛 OR 弥漫性肌筋膜疼痛 OR 弥漫性肌筋膜疼痛综合症

#5 迷走神经刺激 OR 迷走神经

#6 VNS OR taVNS OR ta-VNS OR iVNS

#7 #5 OR #6

#8 #3 AND #4 AND #7
